# Moral distress and associated factors among nurses working in public hospitals in West Arsi Zone, Ethiopia. a cross-sectional study

**DOI:** 10.1186/s12912-026-05143-4

**Published:** 2026-07-31

**Authors:** Lemma Desalegn, Debela Gela, Abdissa Boka

**Affiliations:** 1 Department of Nursing, College of Health Sciences, Arsi University, Asella, Ethiopia; 2 School of Nursing and Midwifery, College of Health Sciences, Addis Ababa University, Addis Ababa, Ethiopia

**Keywords:** Moral distress, Nurses, Public hospitals, Job satisfaction, Working environment, Ethiopia

## Abstract

**Background:**

Moral distress occurs when nurses recognize an ethically appropriate action but feel unable to act due to institutional or systemic constraints. It is a major concern in nursing practice because ethical conflicts, limited autonomy, inadequate resources, and poor organizational support may affect nurses’ well-being and quality of patient care. Evidence on moral distress in Ethiopia remains limited. This study aimed to assess the prevalence of moral distress and factors associated with moral distress among nurses working in public hospitals in the West Arsi Zone, Ethiopia.

**Methods:**

An analytical cross-sectional study was conducted from February 13 to March 2, 2023, among 349 nurses selected by simple random sampling from four public hospitals. Nurses with at least six months of work experience were included, while those unavailable during data collection were excluded. Data were collected using a structured self-administered questionnaire comprising the Moral Distress Scale–Revised (MDS-R) and measures of personal and environmental factors. Data were entered into EpiData version 4.6 and analyzed using SPSS version 26. Moral distress was categorized into low and high levels using the sample mean. Factors associated with moral distress were identified using logistic regression analysis. Statistical significance was declared at *p* < 0.05 with 95% confidence intervals.

**Results:**

Of the 349 eligible nurses, 344 participated (98.5% response rate). The mean (± SD) age of respondents was 31.92 ± 7.0 years, and 194 (55.6%) were female. Overall, 73.1% of nurses experienced high moral distress. Male nurses were less likely to experience high moral distress compared with female nurses (AOR = 0.46; 95% CI: 0.27–0.91). Nurses who were dissatisfied with their jobs had nearly eight times higher odds of experiencing high moral distress (AOR = 7.67 [3.08, 19.12]). Similarly, nurses working in a non-conducive environment had four times higher odds of experiencing high moral distress (AOR = 4.07 [1.92, 8.65]).

**Conclusions:**

A high proportion of nurses experienced moral distress in West Arsi Zone public hospitals. Improving job satisfaction and creating supportive work environments may help reduce moral distress and improve nursing practice.

## Introduction

Moral distress is a psychological response that occurs when an individual is aware of the ethically appropriate action to take but feels powerless to act due to institutional or systemic constraints. First introduced by Jameton in 1984, moral distress has become a widely recognized challenge in healthcare, particularly for nurses who provide frontline care but often have limited decision-making authority [1].

Nurses are especially vulnerable to moral distress because of their continuous and close contact with patients, the hierarchical nature of healthcare systems, and conflicting demands between professional ethics and institutional policies. Repeated exposure to morally distressing situations can erode a nurse’s sense of professional integrity and contribute to emotional exhaustion, reduced job satisfaction, and compromised quality of patient care [2–4].

Globally, several studies have highlighted the widespread nature of moral distress among nurses. In high-income countries such as the United States, Canada, Australia, and Saudi Arabia, prevalence rates of moral distress among nurses have ranged from 72% to over 90% [5–7]. Factors associated with moral distress include inadequate staffing, poor organizational support, lack of autonomy, and ethical conflicts with colleagues or management [8–10].

In low-income countries, however, moral distress is less well studied, and much of the available evidence comes from isolated hospital units such as emergency departments or intensive care settings. In Ethiopia, few studies have explored the magnitude of moral distress across general hospital settings or examined the broader institutional and environmental factors that may contribute to it [11, 12].

Given the increasing burden on nurses in Ethiopia’s public healthcare system, often characterized by high patient loads, resource limitations, and systemic inefficiencies, understanding moral distress in this context is critical. This study adds context-specific evidence by determining the prevalence of moral distress and identifying its associated factors among nurses working in public hospitals in the West Arsi Zone. The findings may provide baseline information for healthcare managers, nursing leaders, and policymakers to develop targeted interventions aimed at improving nurses’ work environments, job satisfaction, ethical practice, and quality of patient care. This study, therefore, aims to assess the prevalence and associated factors of moral distress among nurses working in public hospitals in the West Arsi Zone of southeast Ethiopia.

## Methods

### Study design and setting

An analytical cross-sectional study was conducted among nurses working in public hospitals of West Arsi Zone, Oromia Regional State, Ethiopia, from February 13 to March 2, 2023. The study was conducted in four selected public hospitals: Shashemene Referral Hospital, Malka Oda General Hospital, Dodola General Hospital, and Loke Primary Hospital. West Arsi Zone, with Shashemene as its administrative center, is located 165 km from Addis Ababa and is one of the zones in the Oromia Regional State. The zone has a population of 2,929,894 and comprises various health facilities, including 81 functional health centers and 351 health posts. During the study period, the estimated number of nurses working in the zone was 438. Among these, 375 nurses were working in the four selected hospitals: Shashemene Referral Hospital (113), Malka Oda General Hospital (114), Dodola General Hospital (78), and Loke Primary Hospital (70).

### Study population and eligibility criteria

The source population comprised all nurses working in public hospitals of the West Arsi Zone. The study population included nurses working in all units of the selected hospitals. Nurses who had been actively employed for at least six months were eligible to participate in the study. Nurses providing unpaid (volunteer) services were excluded.

### Sample size determination and sampling procedure

The sample size was determined using the single population proportion formula, assuming a 70.16% prevalence of moral distress, a 95% confidence level, and a 5% margin of error. The calculated initial sample size was 322 nurses. After adding a 10% allowance for possible non-response, the final sample size was increased to 354 nurses. This sample size was also considered adequate to identify factors associated with moral distress using logistic regression analysis.

Where:

**n** = required sample size.

**Z** = standard normal value at 95% confidence level (1.96).

**P** = estimated proportion of moral distress (70.16%).

**d** = margin of error (5%).

Four hospitals were selected from the eight public hospitals in West Arsi Zone using a simple random sampling method. The sampling frame consisted of eligible nurses’ lists obtained from the human resource departments of the selected hospitals. The selected hospitals and their respective numbers of nurses were: Shashemene Referral Hospital (113 nurses), Malka Oda General Hospital (114 nurses), Dodola General Hospital (78 nurses), and Loke Primary Hospital (70 nurses). The required sample size was proportionally allocated to each hospital based on the number of eligible nurses. Finally, study participants were selected from the sampling frame using a simple random sampling (lottery) method.

### Data collection tools and procedures

Data were collected using a structured, self-administered questionnaire consisting of four sections: socio-demographic characteristics (9 items), Moral Distress Scale–Revised (MDS-R) (9 items), environmental factors (21 items covering working environment and perceived organizational support), and personal factors (17 items covering job satisfaction, autonomy, and professional commitment) [8–10].

Each item was measured using a 4-point Likert scale ranging from 1 (strongly disagree) to 4 (strongly agree). The MDS-R and other questionnaire items were adopted from previously validated instruments and relevant literature. Content validity was assessed through expert review, and the questionnaire was pretested among 5% of the sample (18 nurses) at Gambo Hospital to ensure clarity, consistency, and applicability. The internal consistency of the adopted tool was assessed using Cronbach’s alpha, which demonstrated good reliability (α = 0.86). Data were collected by six trained BSc nurses under the supervision of an MSc nursing graduate, and supervisors conducted daily checks for completeness and consistency of the collected data.

### Variables of the study

#### Dependent variable

- Moral distress.

#### Independent variables

- Socio-demographic factors: age, sex, marital status, educational level, monthly income, work experience, unit of assignment, and job position.
- Personal factors: job satisfaction, autonomy, professional commitment.
- Environmental factors: working environment, perceived organizational support.

### Operational definitions

**Moral distress** is a psychological distress resulting from moral constraints or conflicts. It was measured using nine items, and the total score was calculated by summing the responses. The mean score of the scale was used as a cut-off point to categorize moral distress; scores below the mean indicated low moral distress, while scores above the mean indicated high moral distress [8, 13, 14].

**Working environment** a place where nurses collaborated therapeutically with different specialists Based on the mean score of the participants’ responses, it was determined that a working environment was not conducive if it was less than the mean score and a conducive working environment if it was greater than or equal to the mean score [11].

**Perception of organizational support** This describes how the nurses felt about the organizational support they got while working in the hospital. According to the participants’ responses’ mean score, it was deemed to have poor perception if the participant received a score below the mean and good perception if the participant had a score above or equal to the mean [12].

**Job satisfaction** is viewed as a grouping of emotions related to a professional setting, or simply how the nurses feel about their work. It was deemed not satisfied for scores below the mean and satisfied for scores higher than or equal to the mean based on the mean score of the participants’ responses [15].

**Professional autonomy** is the nurse’s discretion or authority to choose in a clinical setting. It was deemed autonomous for responses that were greater than or equal to the mean score, and not autonomous for responses that were less than the mean score [16].

**Professional commitment** refers to the internal attitude that nurses have toward their jobs. According to the participants’ responses, it was deemed committed for scores greater than or equal to the mean and not committed for scores lower than the mean [9, 10].

**Nurses** For this study, a nurse refers to a licensed nursing professional who was actively employed and providing nursing care in any working unit of the selected public hospitals.

### Data processing and analysis

The collected data were checked for completeness and consistency, then coded and entered into EpiData version 3.1 and exported to SPSS version 26 for analysis. Prior to analysis, the distribution of the moral distress score was assessed using histograms, Q–Q plots, and the Shapiro–Wilk test. As the distribution was approximately symmetric and the instrument has no established clinical cut-off point, the sample mean was used to categorize participants into low and high moral distress. Descriptive statistics (frequencies, percentages, means, and standard deviations) were used to summarize the data. Bivariate logistic regression was performed to identify candidate variables (*p* < 0.25) for the multivariable model. Multivariable logistic regression analysis was then used to determine independent predictors of moral distress. Adjusted odds ratios (AOR) with 95% confidence intervals (CI) were calculated, and statistical significance was declared at *p* < 0.05.

### Ethical considerations

Ethical clearance was obtained from the Institutional Review Board (IRB) of Addis Ababa University, College of Health Sciences (protocol No.14/SNM/15). Official permission was secured from the Oromia Health Bureau and the respective hospital administrations. Written informed consent was obtained from each participant prior to data collection. Confidentiality and anonymity were ensured by using coded identifiers and secure data handling. Participation was voluntary, and nurses were informed of their right to withdraw at any point.

## Results

### Sociodemographic characteristics of study respondents

A total of 349 participants were enrolled in this study with a response rate of 98.5%. The mean and standard deviation age of the respondents was 31.92 ± 7.0 years, with the majority of them being 18–35 years of age (276, 76.5%). Among all of the participants, 194(55.6%) of them were females. The majority of the respondents were Oromo 303(86.8%) and almost half of the participants were Muslims 175(50.1%). Out of 349 participants, 293(84.0%) hold a bachelor of degree in nursing profession. The mean and SD year of experience was 7.7 ± 6.9. Among 349 participants, 275(78.8%) of them have 0–10 years of professional experience. The majority of the respondents 249(71.3%) were senior in their position. (Table 1)

**Table 1:** Sociodemographic characteristics of nurses working in West Arsi Zone public hospitals, south-east Ethiopia, March 2023 (*n* = 349)

Variables	Category	Frequency	Percent (%)
Age	18–35	267	76.5
	36–45	59	16.9
	45–60	23	6.6
Sex	Male	155	44.4
	Female	194	55.6
Ethnicity	Oromo	303	86.8
	Amhara	32	9.2
	Tigre	8	2.3
	Others*	6	1.7
Religion	Orthodox	104	29.8
	Muslim	175	50.1
	Protestant	66	18.9
	Others**	4	1.2
Marital status	Single	119	34.1
	Married	215	61.6
	Divorced	11	3.2
	Widowhood	4	1.1
Working unit	Medical ward	52	14.9
	Surgical ward	56	16.0
	Emergency	50	14.3
	ICU	23	6.6
	Paediatrics ward	47	13.5
	Gynaecology	39	11.2
	NICU	34	9.7
	OPD	48	13.8
Educational status	Diploma	43	12.3
	Degree	293	84.0
	Msc	13	3.7
Monthly income	4605-7162.2 ETB	219	62.8
	7162.3-11305 ETB	130	37.2
Experiences(years)	0–10	275	78.8
	11–20	52	14.9
	21–30	22	6.3
Position	Junior	71	20.3
	Senior	249	71.4
	Nurse manager	29	8.3

*(Sidama and Wolaita), ** (Adventist and wakefata)

### Level of moral distress

**Fig. 1:**
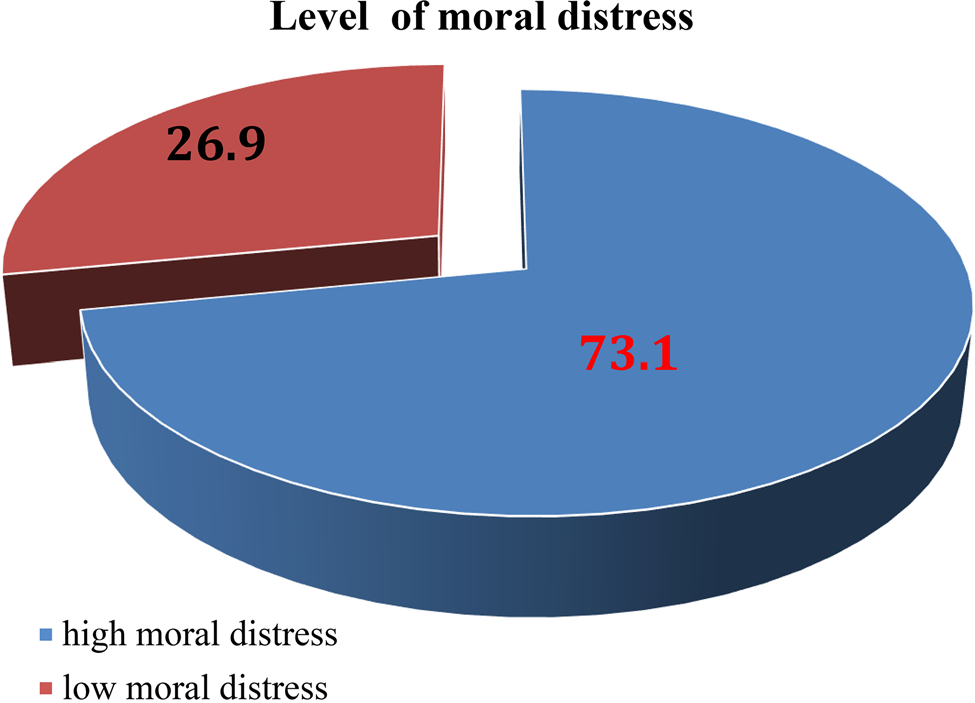
Proportion of moral distress among nurses working in West Arsi Zone public hospitals, south-east Ethiopia, March 2023 (*n* = 349)

Among the 349 nurses who participated in the study, the majority experienced high levels of moral distress, while the remaining participants had low levels of moral distress (Fig. 1). The mean (± SD) moral distress score was 22.8 ± 10.8.

### Personal and environmental factors

Job satisfaction and perception of organizational support were relatively high among nurses, with nearly three-fifths of respondents reporting satisfaction with their jobs and good organizational support. Similarly, slightly more than half of the participants perceived their working environment as conducive. In contrast, a considerable proportion of nurses reported a lack of autonomy and low professional commitment. (Table 2)

**Table 2:** Personal and environmental factors of nurses working in West Arsi Zone public hospitals, south-east Ethiopia, March 2023 (*n* = 349)

Variables	Category	Frequency	Percent (%)
Job Satisfaction	Not satisfied	141	40.4
	Satisfied	208	59.6
Autonomy	Not autonomous	185	53.0
	Autonomous	164	47.0
Professional commitment	Committed	162	46.4
	Not committed	187	53.6
Working environment	Not conducive	170	48.7
	Conducive	179	51.3
Perception of organizational support	Poor perception	143	41.0
	Good perception	206	59.0

### Factors associated with moral distress among nurses

In, bivariate analysis socio demographic characteristics (sex, working unit, monthly salary and years of experience), personal factors (job satisfaction, autonomy, and professional commitment), and Organizational factor (working environment) were had p-value < 0.25 and candidate for the multivariable logistic regression analysis. In, multivariable logistic regression analysis sex, job satisfaction and working environment were associated with moral distress among nurses.

After adjusting for potential confounding variables, male nurses had significantly lower odds of experiencing a high level of moral distress than female nurses (AOR = 0.46, 95% CI: 0.27–0.91, *p* = 0.023). Specifically, the odds of high moral distress among male nurses were 54% lower compared with female nurses.

Nurses who were not satisfied were approximately eight times more likely to experience a high level of moral distress than those who were satisfied. Similarly, nurses working in a non-conducive work environment were approximately four times more likely to experience a high level of moral distress than those working in a conducive environment. (Table 3)

**Table 3:** Factors associated with moral distress among Nurses working in West Arsi zone public hospitals, south east Ethiopia, March, 2023 (*n* = 349)

Variables	Category	Moral distress		COR	AOR
		High	Low		
Sex	Male	100	55	0.46 (0.28, 0.74)*	**0.5 (0.27**, 0.91)*
	Female	155	39	1	1
Working unit	Medical ward	35	17	0.48 (0.19, 1.20) *	0.54 (0.18, 1.64)
	Surgical ward	37	19	0.45 (0.18, 1.12) *	0.36 (0.12, 1.06)
	Emergency	41	9	1.05 (0.38, 2.92)	0.99 (0.30, 3.30)
	ICU	18	5	0.83 (0.24, 2.84)	0.55 (0.12, 2.50)
	Pediatrics ward	32	15	0.49 (0.19, 1.27) *	0.46 (0.15, 1.44)
	Gynecology	24	15	0.37 (0.14, 0.97) *	0.41 (0.13, 1.29)
	NICU	29	5	1.34 (0.41, 4.42)	0.76 (0.19, 3.03)
	OPD	39	9	1	1
Monthly salary	4605-7162.2	168	51	1.63 (1.01, 2.64)	1.59 (0.75, 4.00)
	7162.2-11305	87	43	1	1
Years of experience	0–10	200	75	1.52 (0.61, 3.78)	0.74 (0.21, 2.59)
	11–20	41	11	2.13 (0.71, 6.36) *	1.72 (0.45, 6.58)
	21–30	14	8	1	1
Job satisfaction	Not satisfied	134	7	13.76 (6.13, 30.89)*	**7.67 (3.08**, 19.12)*
	Satisfied	121	87	1	1
Autonomy	Not autonomous	145	40	1.78 (1.10, 2.87)*	1.16 (0.62, 2.15)
	Autonomous	110	54	1	1
Professional commitment	Not committed	129	33	1.89 (1.16, 3.09)*	0.76 (0.39, 1.47)
	Committed	126	61	1	1
Working environment	Not conducive	157	13	9.98 (5.28, 18.89)*	**4.07 (1.92**, 8.65)*
	Conducive	98	81	1	1
Perception of organizational support	Poor perception	130	13	6.48 (3.43, 12.23)*	1.78 (0.77, 4.01)
	Good perception	125	81	1	1

*Adjusted odd ration at (95%) of CI with p value < 0.05 were statistical significance

AOR: Adjusted odd ratio, COR: Crude odd ratio

## Discussion

This study aimed to assess the prevalence of moral distress and identify its associated factors among nurses working in public hospitals of the West Arsi Zone, Oromia Regional State, Ethiopia. This institutional-based analytical cross-sectional study involved 349 nurses working in different units of selected public hospitals. The findings showed that 73.1% of the study participants experienced a high level of moral distress. This finding is comparable to studies conducted in Australia (72%) and Jimma, Ethiopia (70.16%), which also reported a high prevalence of moral distress among nurses using similar study designs and comparable measurement tools [8, 17]. The prevalence observed in this study was higher than that reported in Saudi Arabia (33.8%), Brazil (41.5%), and Iran (24.3%) [7, 18, 19]. These differences may reflect variations in the study settings, study populations, and measurement approaches reported in those studies.

However, the prevalence was lower than that reported in studies conducted in the United States (80%) and northern Ethiopia (83.7%) [13, 20]. The higher prevalence in those studies may be related to differences in participant characteristics, healthcare settings, and assessment methods described by the respective studies.

Female nurses were more likely to experience high moral distress than male nurses. This finding is consistent with studies from Iran [21] and the United States [4], which also reported higher moral distress among female nurses. However, it differs from findings reported in Ethiopian studies [8, 13], suggesting that the relationship between sex and moral distress may vary across different study populations and settings.

Job satisfaction was significantly associated with moral distress. Nurses who were not satisfied with their jobs were nearly eight times more likely to experience high moral distress than those who were satisfied. This finding is consistent with studies conducted in the United States [22], the Philippines [14], southwest Iran [23], the southeastern United States [24], and the Netherlands [25], all of which reported an inverse relationship between job satisfaction and moral distress.

Working environment was also significantly associated with moral distress. Nurses working in an unfavorable work environment were approximately four times more likely to experience high moral distress than those working in a favorable work environment. Similar findings have been reported in studies from Iran [26], Canada [3], Virginia [27], the southeastern United States [24], and Jimma [8], indicating that unfavorable workplace conditions are consistently associated with increased moral distress among nurses. These findings highlight the importance of creating supportive and conducive work environments to reduce moral distress among nurses. Furthermore, unfavorable working conditions induce moral distress and burnout. This also in turn affects patient centered care, health team communication, patient’s family, community, and health institution. The high prevalence of moral distress and its association with sex, job satisfaction, and working environment indicate the need for targeted organizational interventions. Hospital administrators and policymakers should consider nurses’ sociodemographic characteristics when developing strategies to reduce moral distress. Improving job satisfaction, strengthening supportive leadership, creating conducive work environments, and providing ethics support programs may help reduce moral distress, promote nurses’ well-being, and improve the quality of patient care.

### Limitations of the study

This study has some limitations. First, the analytical cross-sectional design does not permit causal inference. Second, differences in respondents’ understanding of moral distress may have influenced the findings. Finally, the study was limited to public hospitals; therefore, the findings may not be generalizable to nurses working in health centers or private hospitals.

## Conclusion and recommendation

### Conclusion

Moral distress was highly prevalent among nurses working in public hospitals in West Arsi Zone, affecting nearly three-quarters of the study participants. The findings indicate that both individual and workplace-related factors contribute to moral distress, particularly sex, job satisfaction, and the working environment. This highlights moral distress as a significant occupational and organizational concern that may adversely affect nurses’ well-being and the quality of patient care. Therefore, healthcare institutions should prioritize strategies aimed at improving the work environment and enhancing job satisfaction to mitigate moral distress among nurses.

### Recommendation

Based on the study findings, hospital administrators and managers should improve the working environment, enhance job satisfaction, and strengthen organizational support to reduce moral distress among nurses. Public hospitals in the West Arsi Zone should give particular attention to female nurses by improving working conditions and providing training, educational programs, and emotional support to address factors contributing to moral distress. The Ethiopian Nursing Association should also implement initiatives to reduce moral distress among nurses. Furthermore, future researchers are encouraged to conduct mixed-methods studies to explore the underlying causes of moral distress and build on the findings of the present study.

## Data Availability

No datasets were generated or analysed during the current study.

## References

[1] Morley G, Ives J, Bradbury-Jones C, Irvine F. What is ‘moral distress’? A narrative synthesis of the literature. Nurs Ethics. 2019;26(3):646–62. 28990446 10.1177/0969733017724354 PMC6506903

[2] Morley G. What is moral distress in nursing? How, can and should we respond to it? J Clin Nurs. 2018;27(19–20):3443–5. 29495084 10.1111/jocn.14332 PMC6175312

[3] Austin W. Moral distress and the contemporary plight of health professionals. HEC Forum. 2012;24(1):27–38. 22441996 10.1007/s10730-012-9179-8

[4] O’Connell CB. Gender and the experience of moral distress in critical care nurses. Nurs Ethics. 2015;22(1):32–42. 24482261 10.1177/0969733013513216

[5] Young A, Froggatt K, Brearley SG. Powerlessness’ or ‘doing the right thing’ – Moral distress among nursing home staff caring for residents at the end of life: An interpretive descriptive study. Palliat Med. 2017;31(9):853–60. 28659023 10.1177/0269216316682894

[6] Emmamally W, Chiyangwa O. Exploring moral distress among critical care nurses at a private hospital in Kwa-Zulu Natal, South Africa. South Afr J Crit Care. 2020;36(2):104–8. 10.7196/SAJCC.2020.v36i2.435 PMC9045500 35493280

[7] Almutairi AF, Salam M, Adlan AA, Alturki AS. Prevalence of severe moral distress among healthcare providers in Saudi Arabia. Psychol Res Behav Manag. 2019;12:107–15. 30804690 10.2147/PRBM.S191037 PMC6375112

[8] Beyaffers HA, Woldetsadik MT, Gizaw AB. Predictors of moral distress among nurses working in Jimma University Medical Center, South West Ethiopia. Front Nurs. 2021;7(4):369–77.

[9] Gary J, Blau. The measurement and prediction of career commitment. J Occup Psychol. 1985;58(1983):277–88.

[10] Wang L, Tao H, Ellenbecker CH, Liu X. Job satisfaction, occupational commitment and intent to stay among Chinese nurses: A cross-sectional questionnaire survey. J Adv Nurs. 2012;68(3):539–49. 21722170 10.1111/j.1365-2648.2011.05755.x

[11] Tian X, Jin Y, Chen H, Jiménez-Herrera MF. Instruments for detecting moral distress in clinical nurses: a systematic review. Inq (United States). 2021;58. 10.1177/0046958021996499 PMC8743918 33771048

[12] Robaee N, Atashzadeh-Shoorideh F, Ashktorab T, Baghestani A, Barkhordari-Sharifabad M. Perceived organizational support and moral distress among nurses. BMC Nurs. 2018;17(1):1–7. 29344004 10.1186/s12912-017-0270-y PMC5763610

[13] Berhie AY, Tezera ZB, Azagew AW. Moral distress and its associated factors among nurses in northwest amhara regional state referral hospitals, northwest ethiopia. Psychol Res Behav Manag. 2020;13:161–7. 32110124 10.2147/PRBM.S234446 PMC7037049

[14] Watson-Subia MM. Moral Distress And Job Satisfaction Of Nurses In Private Hospitals. Int J Nurs Heal Sci. 2019;5(2):1–5.

[15] Martins H. Teresa proença. Wp471.Pdf. 2012;(October).

[16] Cho E, Choi M, Kim EY, Yoo IY, Lee NJ. Construct validity and reliability of the korean version of the practice environment scale of nursing work index for korean nurses. J Korean Acad Nurs. 2011;41(3):325–32. 21804341 10.4040/jkan.2011.41.3.325

[17] Prentice TM, Gillam L, Davis PG, Janvier A. Always a burden? Healthcare providers’ perspectives on moral distress. Arch Dis Child Fetal Neonatal Ed. 2018;103(5):F441–5. 28970316 10.1136/archdischild-2017-313539

[18] Laurs L, Blaževičienė A, Capezuti E, Milonas D. Moral Distress and Intention to Leave the Profession: Lithuanian Nurses in Municipal Hospitals. J Nurs Scholarsh. 2020;52(2):201–9. 31837105 10.1111/jnu.12536

[19] Tajalli S, Rostamli S, Dezvaree N, Shariat M, Kadivar M. Moral distress among iranian neonatal intensive care units’ health care providers: A multi-center cross sectional study. J Med Ethics Hist Med. 2021;14(12):5–9. 35035800 10.18502/jmehm.v14i12.7667 PMC8696547

[20] Corley MC. Nurse moral distress. Nurs Ethics [Internet]. 2002;9(6):636–50. Available from: http://journals.sagepub.com/doi/10.1191/0969733002ne557oa. 10.1191/0969733002ne557oa 12450000

[21] Babamohamadi H, Bakuei Katrimi S, Paknazar F. Moral distress and its contributing factors among emergency department nurses: A cross-sectional study in Iran. Int Emerg Nurs [Internet]. 2021;56(January):100982. Available from: 10.1016/j.ienj.2021.100982 10.1016/j.ienj.2021.100982 33714726

[22] Davidson JE, Doran N, Petty A, Arellano DL, Henneman EA, Hanneman SK, et al. Survey of nurses’ experiences applying the joint commission’s medication management titration standards. Am J Crit Care [Internet]. 2021;30(5):365–74. Available from: https://aacnjournals.org/ajcconline/article/30/5/365/31549/Survey-of-Nurses-Experiences-Applying-The-Joint. 10.4037/ajcc2021716 34467387

[23] Molazem Z, Bagheri L, Najafi Kalyani M. Evaluation of the Moral Distress Intensity and Its Relationship with the Quality of Work Life among Nurses Working in Oncology Wards in Shiraz, Southwest of Iran. Biomed Res Int. 2022;2022:3–6. 10.1155/2022/7977039 PMC9626198 36330461

[24] Asgari S, Shafipour V, Taraghi Z, Yazdani-Charati J. Relationship between moral distress and ethical climate with job satisfaction in nurses. Nurs Ethics [Internet]. 2019;26(2):346–56. Available from: https://journals.sagepub.com/doi/10.1177/0969733017712083. 10.1177/0969733017712083 28718349

[25] de V AJ, F AL, S A. D.L. W. Determinants of moral distress in daily nursing practice: a cross sectional correlational questionnaire survey. Int J Nurs Stud [Internet]. 2013;50(1):100–8. Available from: http://www.embase.com/search/results. 10.1016/j.ijnurstu.2012.08.017 22989404

[26] Bayat M, Shahriari M, Keshvari M. The relationship between moral distress in nurses and ethical climate in selected hospitals of the Iranian social security organization. J Med Ethics Hist Med. 2019;12(8):1–16. 32328221 10.18502/jmehm.v12i8.1339 PMC7166247

[27] Sheppard KN, Runk BG, Maduro RS, Fancher M, Mayo AN, Wilmoth DD, et al. Nursing Moral Distress and Intent to Leave Employment During the COVID-19 Pandemic. J Nurs Care Qual. 2022;37(1):28–34. 34538814 10.1097/NCQ.0000000000000596 PMC8607915

